# Comment on “The relationship of FricTest® responses with an urticaria activity score, urticaria control test and quality of life scales in patients with symptomatic dermographism”^؆^

**DOI:** 10.1016/j.abd.2025.501285

**Published:** 2026-01-15

**Authors:** Prajnasini Satapathy, Rachana Mehta, Ranjana Sah

**Affiliations:** a Center for Global Health Research, Saveetha Medical College and Hospital, Saveetha Institute of Medical and Technical Sciences, Chennai, Tamil Nadu, India; b Clinical Microbiology, RDC, Manav Rachna International Institute of Research and Studies, Faridabad, Haryana, 121004, India; c Dr. D. Y. Patil Medical College Hospital and Research Centre, Dr. D. Y. Patil Vidyapeeth (Deemed-to-be-University), Pimpri, Maharashtra, India

Dear Editor,

We read with great interest the study by Kıraç et al. examining the associations between FricTest® response levels and clinical assessment tools in symptomatic dermographism.^1^ Their work contributes to a practical effort to quantify disease activity and patient-reported burden using a tool with potential for broad clinical utility. The authors’ findings demonstrated a moderate correlation between changes in the 4.5 mm and 4 mm FricTest® responses and validated scales, such as the urticaria control test (UCT), urticaria activity score (UAS), and dermatology life quality index (DLQI), suggesting that higher-intensity wheal reactivity may be more clinically informative.

However, key aspects of the statistical approach may limit the confidence in the strength and applicability of these associations. The analysis relied on Spearman correlation coefficients but did not report confidence intervals, which restricts the interpretability of the observed effect sizes. More importantly, correlation does not imply predictive capacity. Without regression modeling to adjust for baseline disease severity or demographic covariates, it remains unclear whether changes in FricTest® values are independently associated with treatment response or simply reflect regression to the mean. This limitation is not merely a technical issue. In a clinical setting, dermatologists must decide whether repeating the FricTest® meaningfully informs therapeutic adjustments or patient counseling. Without understanding its added predictive value over conventional scales (e.g., UCT), the test’s standalone contribution remains unclear.

Furthermore, the lack of correlation between FricTest® changes and visual analog scale (VAS) scores for itch and burning raises questions about the pathophysiological specificity of this measurement. If the test reflects mechanical wheel responsiveness but not subjective symptom severity, its role may be limited to diagnostic rather than longitudinal monitoring contexts.^2^, ^3^ This disconnect merits closer evaluation, particularly as symptomatic dermographism is fundamentally a patient-experienced condition.

Despite these concerns, this study adds value by suggesting that lower-pressure stimuli (3.5 and 3 mm) do not correlate well with validated scales. This narrows the clinically useful thresholds and may help streamline test administration protocols. This also reinforces the fact that excessive granularity in provocation testing may introduce statistical noise without clinical gain.

We commend the authors for integrating disease-specific and quality-of-life instruments in a unified analysis and for contributing data on a relatively under-investigated diagnostic tool. Future research should focus on validating these findings through multivariable models and exploring whether FricTest® changes the forecast of relapse or remission when used alongside standard scales.

## ORCID IDs

Prajnasini Satapathy: 0009-0007-1805-775X

Rachana Mehta: 0009-0002-9627-5326

Ranjana Sah: 0009-0009-8332-6391

## Declaration of Generative AI and AI-assisted technologies in the writing process

Generative AI tools, including Paperpal and ChatGPT-4o, were utilized solely for language refinement, grammar enhancement, and stylistic refinement. These tools had no role in the conceptualization, data analysis, interpretation of results, or substantive content development of this manuscript. All intellectual contributions, data analysis, and scientific interpretations remain the sole work of the authors. The final content was critically reviewed and edited to ensure accuracy and originality. The authors take full responsibility for the accuracy, originality, and integrity of the work presented.

## Financial support

None declared.

## Research data availability

Does not apply.

## Authors' contributions

**Prajnasini Satapathy:** Conceptualization, Methodology, Writing - original draft, Writing - review & editing. **Rachana Mehta:** Writing - original draft, Writing - review & editing. **Ranjana Sah:** Validation, Supervision, Project administration, Writing - original draft, Writing - review & editing.

## Conflicts of interest

None declared.
